# Supplementary material for: Observation of giant dipole moments of interlayer excitons via layer engineering
Source: Nat Commun. 2025 Nov 27;16:10661. doi: 10.1038/s41467-025-65683-2 (PMC12661042; doi:10.1038/s41467-025-65683-2)
Supplement: Supplementary file 1 — Supplementary Information [file 41467_2025_65683_MOESM1_ESM.pdf]

Supplementary materials for "Observation of Giant Dipole Moments of Interlayer Excitons via Layer Engineering"

Jiasen Zhu[1,†], Ting Liang[1,†], Fuhuan Shen[1,†*], Zefeng Chen[1,2*], Jianbin Xu[1,3*]

1. Department of Electronic Engineering, and Materials Science and Technology Research Center, The Chinese University of Hong Kong, Shatin, N.T., Hong Kong SAR, P. R. China.
2. School of Electronic and Information Engineering, South China Normal University, P. R. China
3. Shenzhen Research Institute, The Chinese University of Hong Kong, Shenzhen, 518057, P. R. China

† These authors contributed equally

* Corresponding Author:

Emails: fhshenbbd@gmail.com (F. H. Shen), zefengchen@m.scnu.edu.cn (Z. F. Chen), jbxu@ee.cuhk.edu.hk (J. B. Xu)

Supplementary Note S1. Calculate the electric field in the HS

In a parallel plate capacitor with a single slab of material in between, the electric field can be defined to be the ratio between the voltage difference and the distance between the parallel plates[1]. The electric field reported in this manuscript is calculated by:

$$E_{hs} = (V_{tg} - V_{bg}) \,/\, \left(t_{h-BN} * \left(\frac{\varepsilon_{h-BN}}{\varepsilon_{hs}}\right) + t_{TMDC}\right) \tag{1}$$

where V_{tg} is the top gate voltage, V_{bg} is the bottom gate voltage, t_{h-BN} is the sum of the thickness of the top and bottom h-BN layers, and t_{TMDC} is the sum of the thickness of the WS_2 and InSe layers. ε_{h-BN}=3.7 are the dielectric constants for h-BN, the dielectric constant of the heterostructure (ε_{hs}) was determined by taking the weighted average (weighted by layer thickness) of the dielectric constant of the TMD and hBN layers. This is calculated to be:

$$\varepsilon_{\mathrm{hs}} = \left\{\left(t_{\mathrm{WS_2}} * \varepsilon_{\mathrm{WS_2}}\right) + \left(t_{InSe} * \varepsilon_{InSe}\right)\right\} / \left(t_{\mathrm{WS_2}} + t_{InSe}\right) \tag{2}$$

where$t_{\mathrm{WS_2}}$, t_{InSe} and t_{hBN} are the thickness of the WS_2, InSe and total hBN layers respectively.

Along with the electric field, there will be electrostatic doping due to the gate voltages. We define the carrier density doping due to the two gates in the intrinsic regime to be:

$$n = \frac{C_{tg}V_{tg}}{e} + \frac{C_{bg}V_{bg}}{e} - n_0 \tag{3}$$

where $C_{tg} = \varepsilon_{\mathrm{h}-BN}/t_{top}$ and $C_{bg} = \varepsilon_{\mathrm{h}-BN}/t_{bot}$ are the capacitances per unit area for the top and bottom gates, e is electron charge, and $\mathrm{n_0}$ is the density of in-gap states needed to be filled before filling the conduction band. In order to avoid electrostatic doping of the TMDs, we use:

$$V_{tg} = -\alpha V_{bg} \tag{4}$$

with $\alpha = t_{top}/\ t_{bottom}$=1.164, where t_{top}=42.5 nm and t_{bottom}=36.5 nm are the top and bottom h-BN thicknesses respectively. When Eq. S4 is inserted into Eq. S3 we get no change in doping due to the electrostatic gates[2]. This gate configuration is defined to be only changing the electric field without changing the doping

level in the TMD layers. The thickness of the h-BN layers was measured using an atomic force microscope. (see Supplementary Fig. **S7**). For detailed fitting parameters used in the calculation of the electric field, refer to Supplementary **Table S1**.

Supplementary Note S2. Methods for DFT and GW-BSE calculations

In this work, the quasiparticle band structure, optical spectra, and exciton wavefunctions were obtained from DFT-based calculations performed using the Vienna Ab initio Simulation Package (VASP, version 6.5.1), employing the projector augmented wave (PAW) method [3] and a plane-wave basis set [4]. The electronic exchange-correlation interaction was described using the Perdew–Burke–Ernzerhof (PBE) functional [5] under the generalized gradient approximation (GGA). At DFT-level calculations, an energy cutoff of 520 eV and a $15 \times 15 \times 1$ k-point grid with Gamma-centered were used. In structural optimizations of InSe and WS_2 with varying numbers of layers, energy and force convergence criteria of 10^{-10} eV and 10^{-4} eV Å^{-1} were employed, respectively. To prevent periodic interactions in the z-direction, a vacuum layer of 30 Å thickness. The D3 correction [6]is applied to characterize the van der Waals interactions between interlayers.

For the quasiparticle calculations, PBE+D3–derived eigenvalues and wavefunctions were used as inputs for the full-frequency-dependent GW calculations [7]performed at the G_0W_0 level. An energy convergence threshold of 10^{-8}eV was enforced, using a 420 eV cutoff and 0.05 eV Gaussian smearing of the partial occupancies to accurately compute the k−derivatives of the orbitals required for the quasiparticle band-structure calculations. GW and BSE calculations were performed using PAW pseudopotentials specifically optimized for the GW method [7]. In the dielectric and self-energy evaluations, unoccupied bands up to ten times the number of occupied valence bands were included, and the energy cutoff of the response function was 100 eV. Upon G_0W_0 calculations, band structures were interpolated via the WANNIER90 package [8]. The imaginary part of the complex dielectric function and all-optical excitations were then obtained by solving the Bethe–Salpeter equation [9], employing the 12 highest valence and 19 lowest conduction bands to extract excitation energies and oscillator strengths. Here, all exciton wavefunctions correspond to the first bright exciton. The visualization of exciton wavefunctions was using VESTA software [10].

In our first-principle calculations (DFT and GW-BSE), interlayer coupling and hybridization are physical

results intrinsically embedded within the computational framework, rather than manually introduced parameters. When calculating the exciton wavefunctions in multilayer InSe/WS_2, the Hamiltonian inherently includes all intra-layer and inter-layer hopping terms. Therefore, the distribution of the electron/hole wavefunction is a result of interlayer hybridization and Coulomb attraction. Furthermore, we employed the D3 correction ([cite: J. Chem. Phys. 2010, 132, 154104]) to ensure the physical accuracy of the interlayer distances, providing a reliable foundation for the calculation.

Vtg (V)	Top BN Thickness (nm)	Bottom BN Thickness (nm)	Fz (V/nm)	Number of WS_2 layer	Number of InSe layer	ε_{ws2}	ε_{InSe}	ε_{hBN}	ε_{hs}
1	26.5	31	-0.0190	2	3	7.3	7	3.7	7.111
1	42.5	36.5	-0.0119	2	4	7.3	7	3.7	7.091
1	42.5	36.5	-0.0119	2	5	7.3	7	3.7	7.078
1	22	41	-0.0238	2	6	7.3	7	3.7	7.068
1	22	41	-0.0224	3	6	7.3	7	3.7	7.091

Table S1. Fitting parameters for the calculation for the electric field.

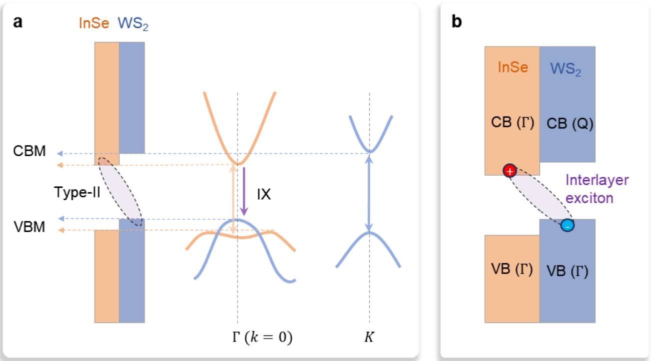

Figure S1. (**a**) The schematic band structure of WS2/InSe heterostructure. (**b**) Simplified model to illustrate the interlayer transition between CBM of InSe and VBM of WS_2.

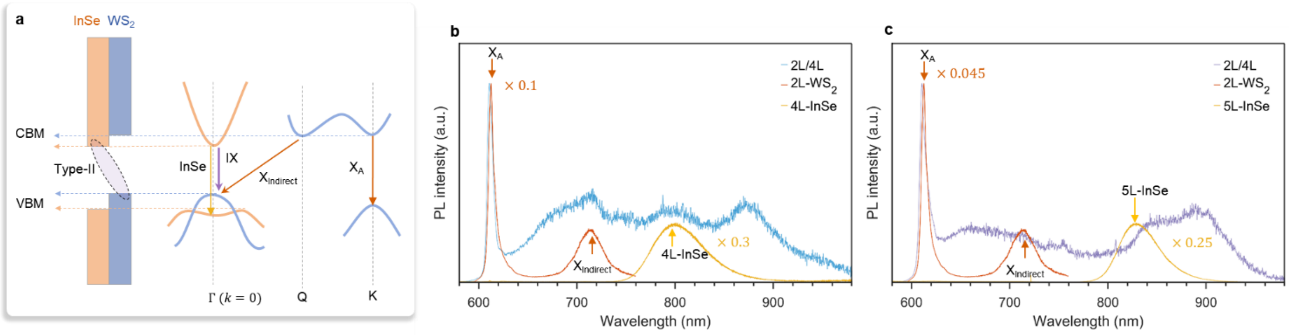

Figure S2. (**a**) Schematic band structure to illustrate the intralayer and interlayer exciton transitions. (**b**) Comparison of PL spectra for 2L/4L heterostructure with the isolated 2L-WS_2 and 4L-InSe.

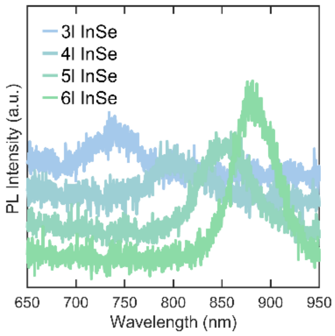

Figure S3. Photoluminescence (PL) spectra of several nL-InSe samples, with the number of layers (L) ranging from 3 to 6.

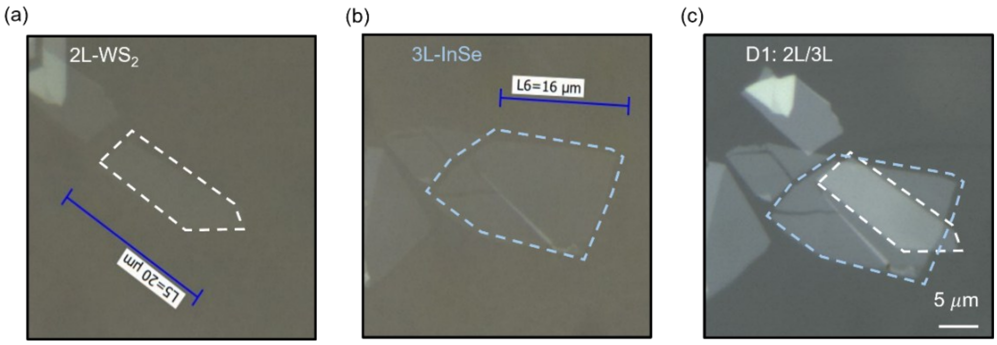

Figure S4. Optical images of isolated TMDC layers and the heterojunction of the Device D1. (a) Isolated WS_2 layer. (b) Isolated InSe layer. (c) Heterojunction formed by stacking WS2 and InSe.

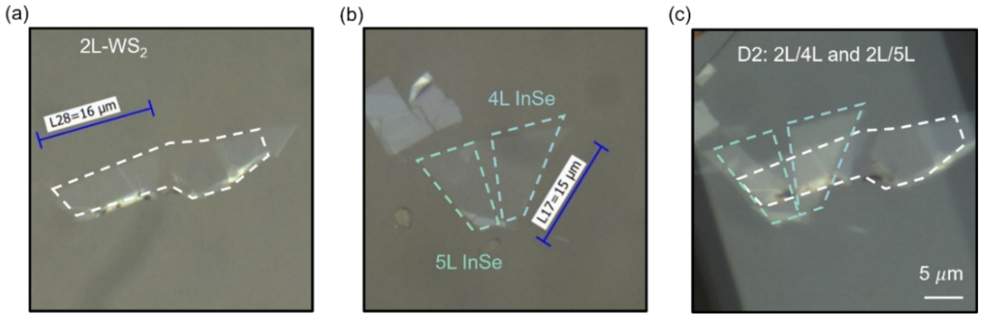

Figure S5. Optical images of isolated TMDC layers and the heterojunction of the Device D2. (a) Isolated WS_2 layer. (b) Isolated InSe layer. (c) Heterojunction formed by stacking WS2 and InSe.

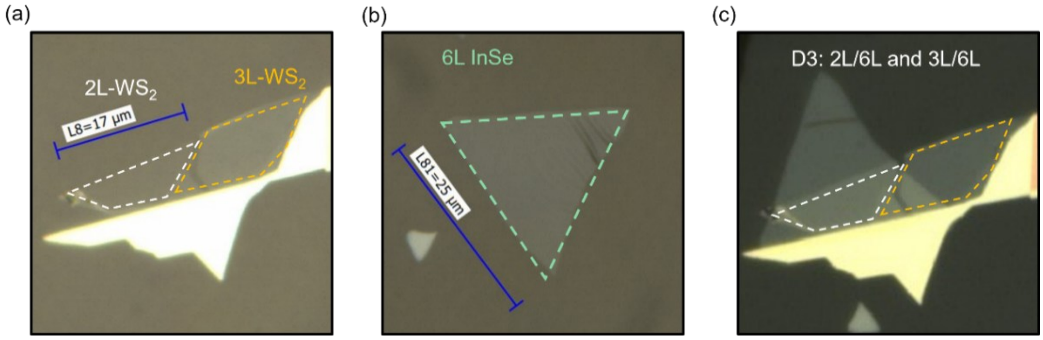

Figure S6. Optical images of isolated TMDC layers and the heterojunction of the Device D3. (a) Isolated WS2 layer. (b) Isolated InSe layer. (c) Heterojunction formed by stacking WS2 and InSe.

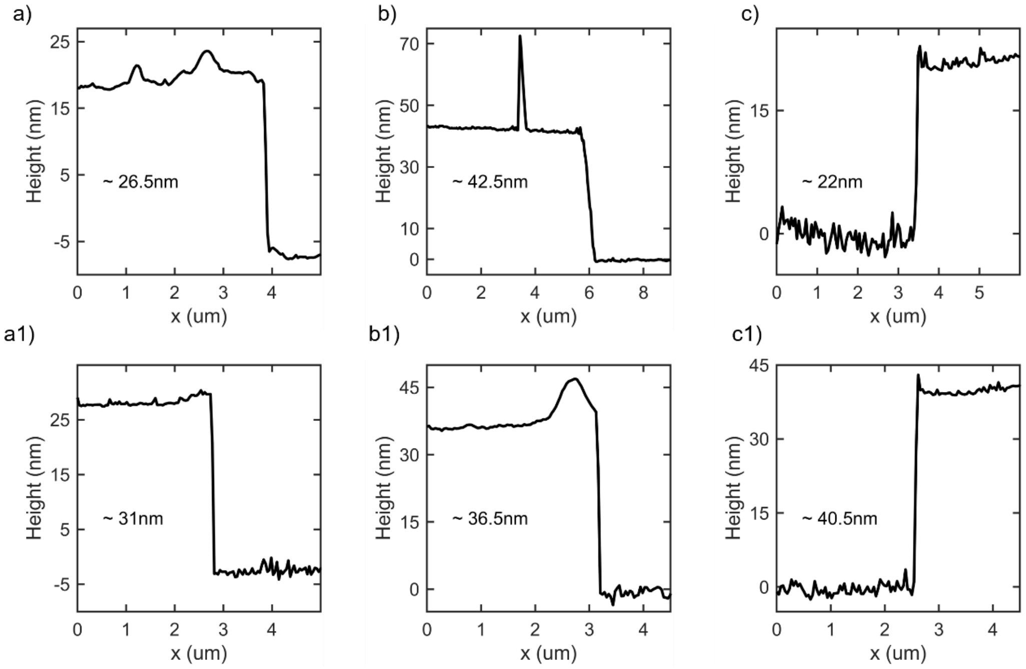

Figure S7. Atomic force microscopy (AFM) of h-BN in Devices D1, D2, and D3. (**a, a1**) AFM measurements of the height of the top and bottom h-BN in Device D1 (2L/3L). (**b, b1**) AFM measurements of the height of the top and bottom h-BN in Device D2 (2L/4L and 2L/5L). (**c, c1**) AFM measurements of the height of the top and bottom h-BN in Device D3 (2L/6L and 3L/6L).

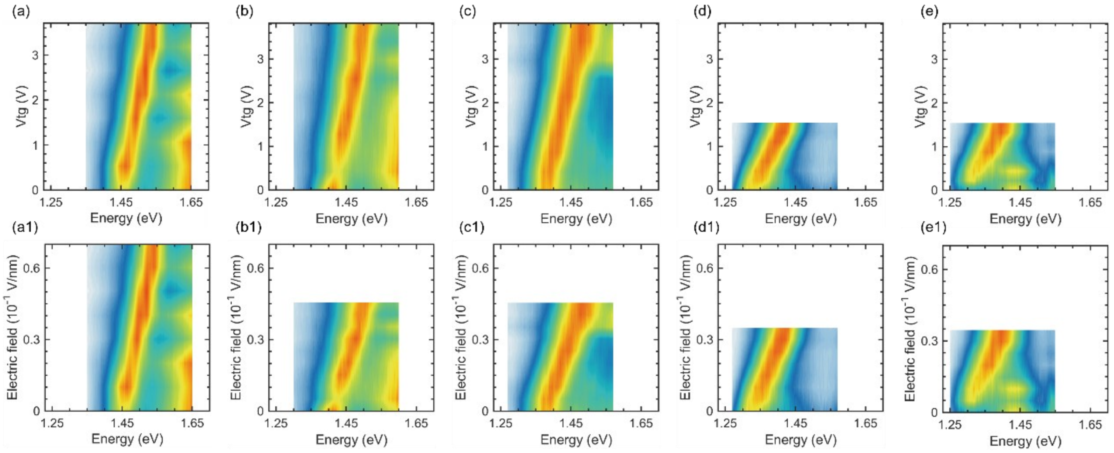

Figure S8. (**a-e**) Pseudocolor map of PL intensity as a function of applied voltage at selected thickness combinations. (**a**) Device D1 (2L/3L). (**b**) Device D2 (2L/4L). (**c**) Device D2 (2L/5L). (**d**) Device D3 (2L/6L). (**e**) Device D3 (3L/6L). (**a1-e1**) Pseudocolor map of PL intensity as a function of applied electric field at selected thickness combinations. (**a1**) Device D1 (2L/3L). (**b1**) Device D2 (2L/4L). (**c1**) Device D2 (2L/5L). (**d1**) Device D3 (2L/6L). (**e1**) Device D3 (3L/6L).

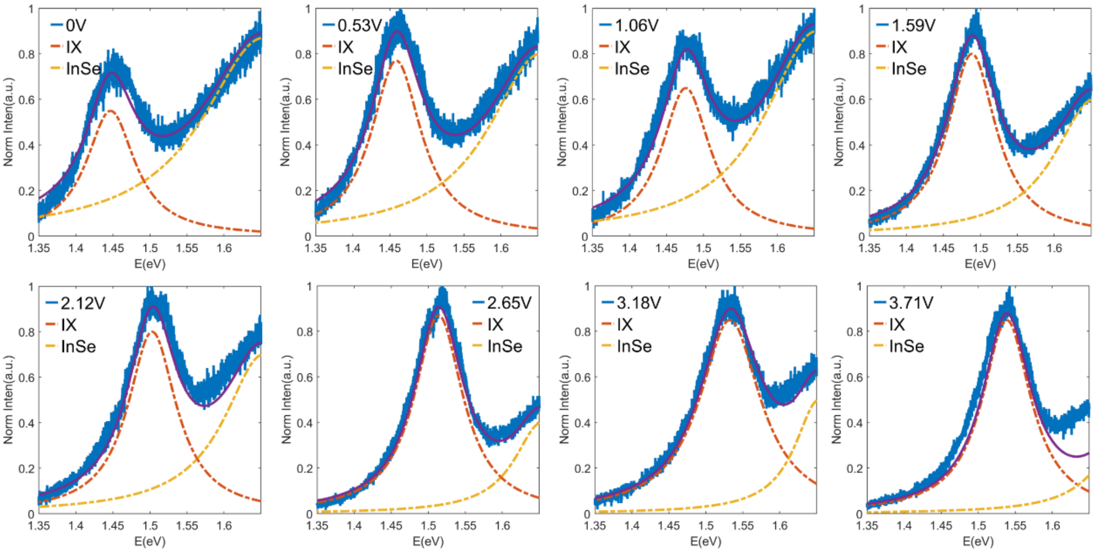

Figure S9. The Lorentz fitting of the PL spectra for Device D1 (2L/3L) shown in **Figure 2d** of the main text. (nV: PL spectra as a function of applied electric field; IX: Interlayer exciton of the HSs; InSe: Intralayer exciton of InSe)

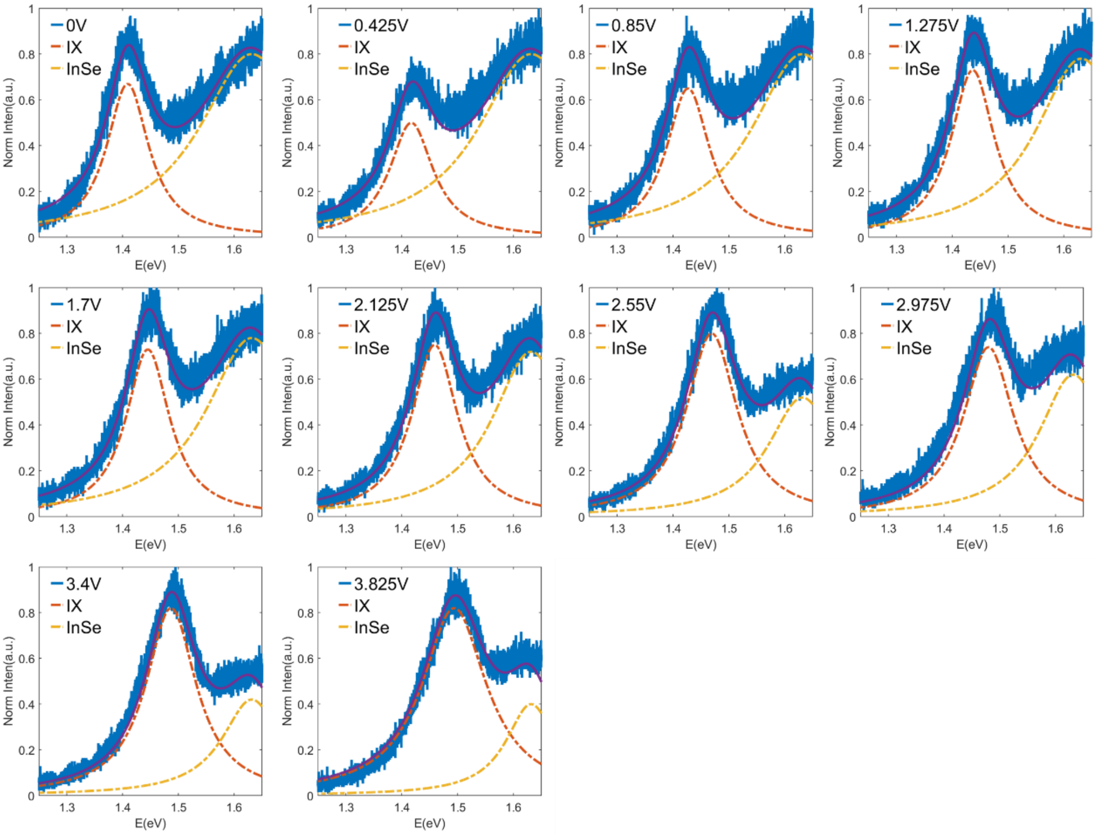

Figure S10. The Lorentz fitting of the PL spectra for Device D2 (2L/4L) shown in **Figure 2d** of the main text. (nV: PL spectra as a function of applied electric f1ield; IX: Interlayer exciton of the HSs; InSe: Intralayer exciton of InSe)

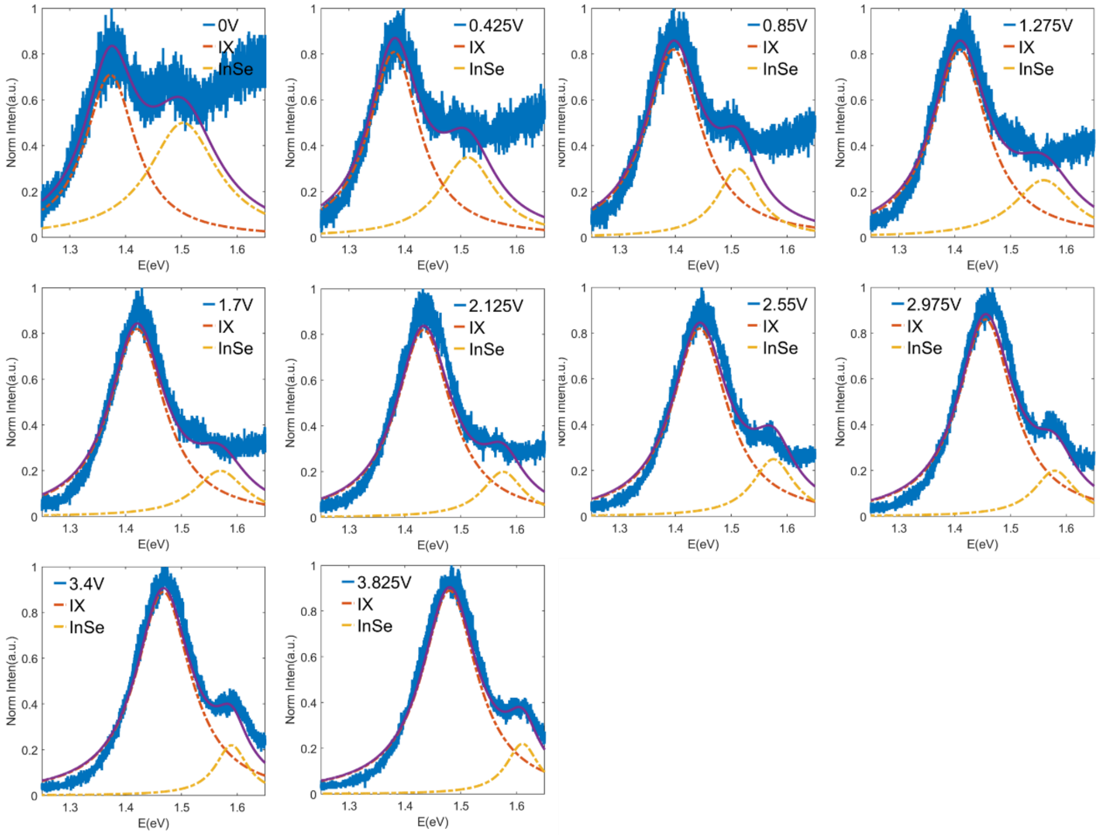

Figure S11. The Lorentz fitting of the PL spectra for Device D2 (2L/5L) shown in **Figure 2d** of the main text. (nV: PL spectra as a function of applied electric f1ield; IX: Interlayer exciton of the HSs; InSe: Intralayer exciton of InSe)

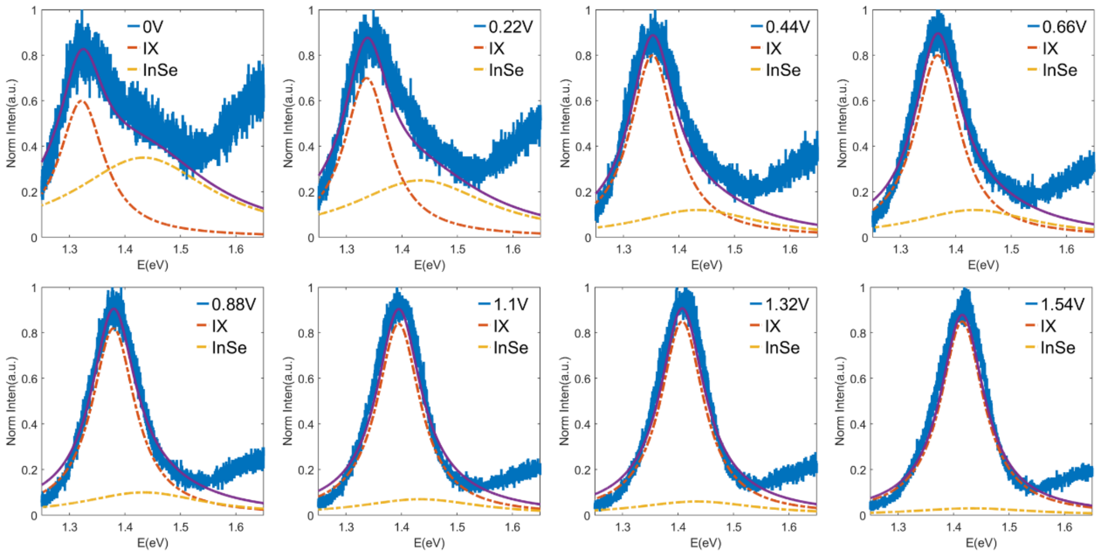

Figure S12. The Lorentz fitting of the PL spectra for Device D3 (2L/6L) shown in **Figure 2d** of the main text. (nV: PL spectra as a function of applied electric f1ield; IX: Interlayer exciton of the HSs; InSe: Intralayer exciton of InSe)

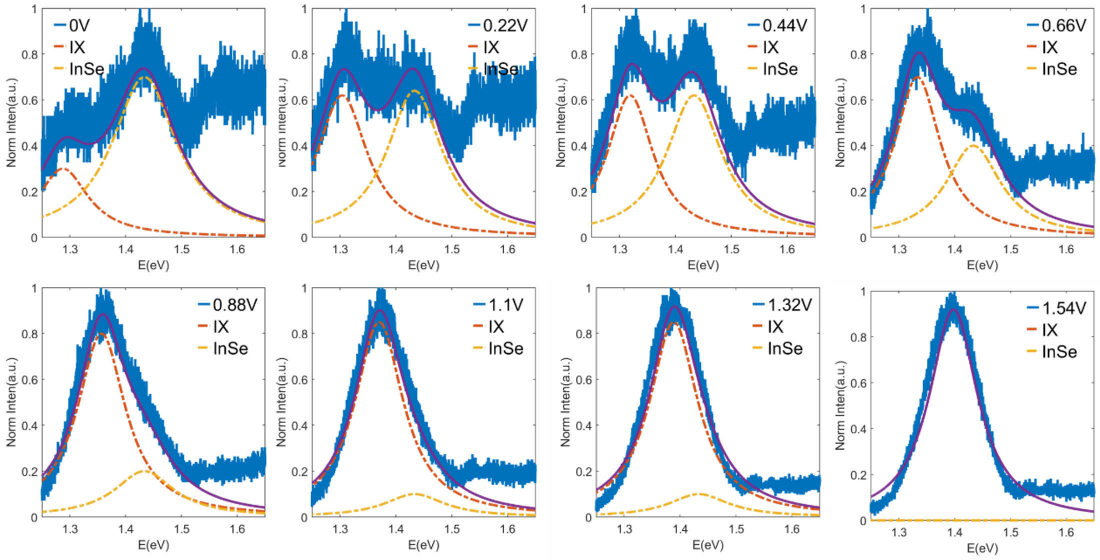

Figure S13. The Lorentz fitting of the PL spectra for Device D3 (3L/6L) shown in **Figure 4c** of the main text. (nV: PL spectra as a function of applied electric f1ield; IX: Interlayer exciton of the HSs; InSe: Intralayer exciton of InSe)

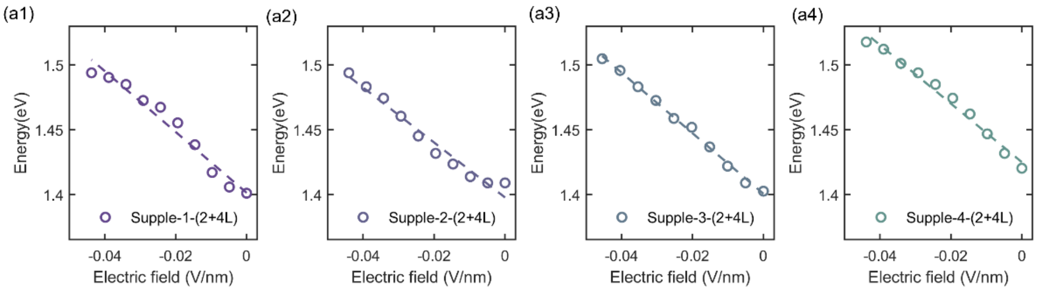

Figure S14. Electric field dependence of interlayer exciton energies in 2/4L structures. Circles represent experimental data, while curves indicate fitting results. Panels (**a1–a4**) correspond to a series of supplementary devices with the same layer combination.

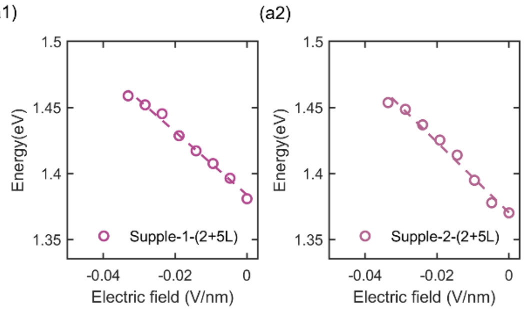

Figure S15. Electric field dependence of interlayer exciton energies in 2/5L structures. Circles represent experimental data, while curves indicate fitting results. Panels (**a1–a2**) correspond to a series of supplementary devices with the same layer combination.

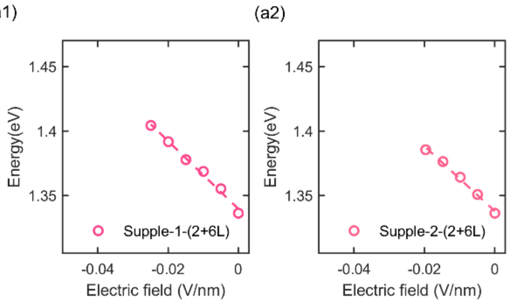

Figure S16. Electric field dependence of interlayer exciton energies in 2/6L structures. Circles represent experimental data, while curves indicate fitting results. Panels (**a1–a2**) correspond to a series of supplementary devices with the same layer combination.

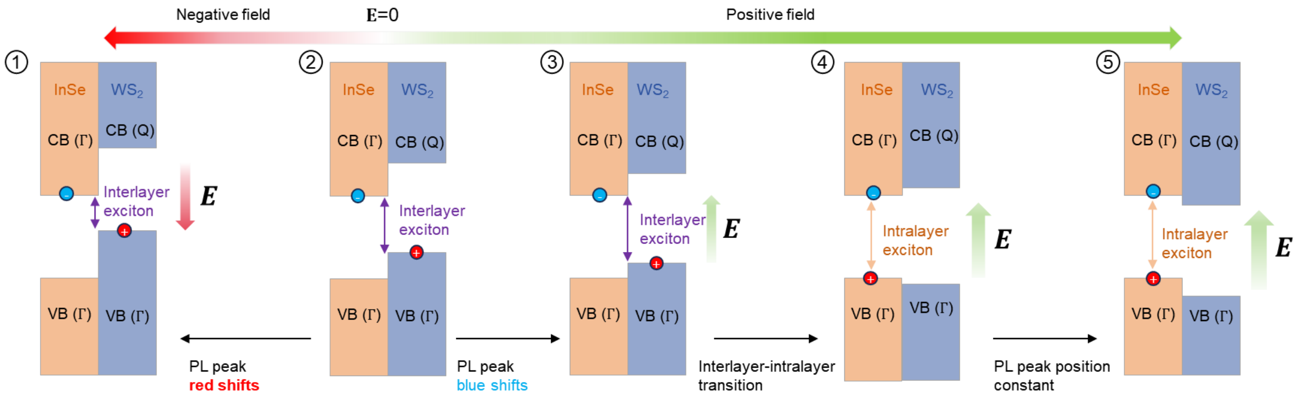

Figure S17. Schematic illustration of the evolution of band structures for heterostructure with the applied electric field.

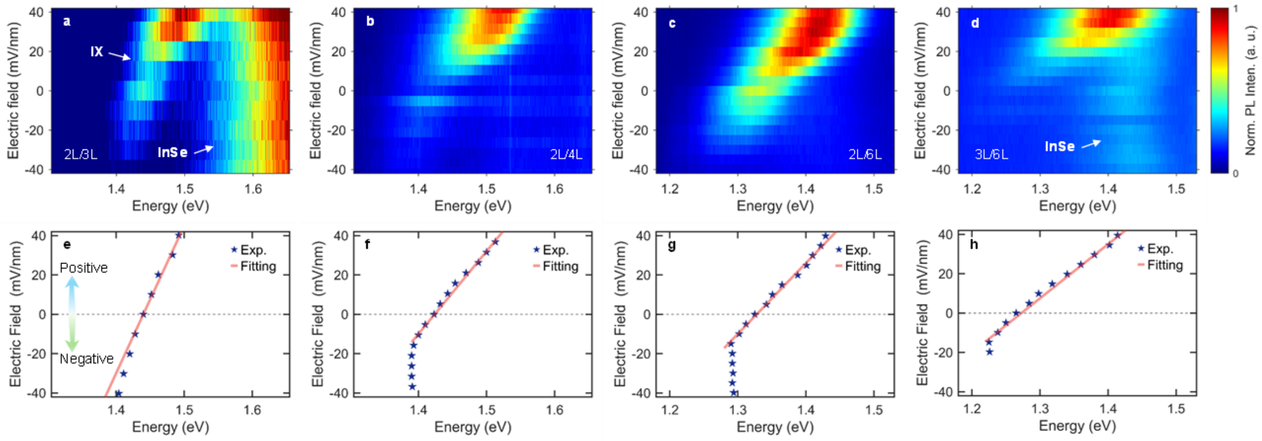

Figure 18. Evolution of IX PL with the electric field for 2L/3L (**a**), 2L/4L (**b**), 2L/6L (**c**), and 3L/6L (**d**) heterostructures where mL/nL represents that heterostructure is composed of mL-WS_2 and nL-InSe. (**e-h**) Corresponding IX peak positions extracted from (**a-d**). Red line is the guide for the eye for the linear dispersion of IX with electric field.

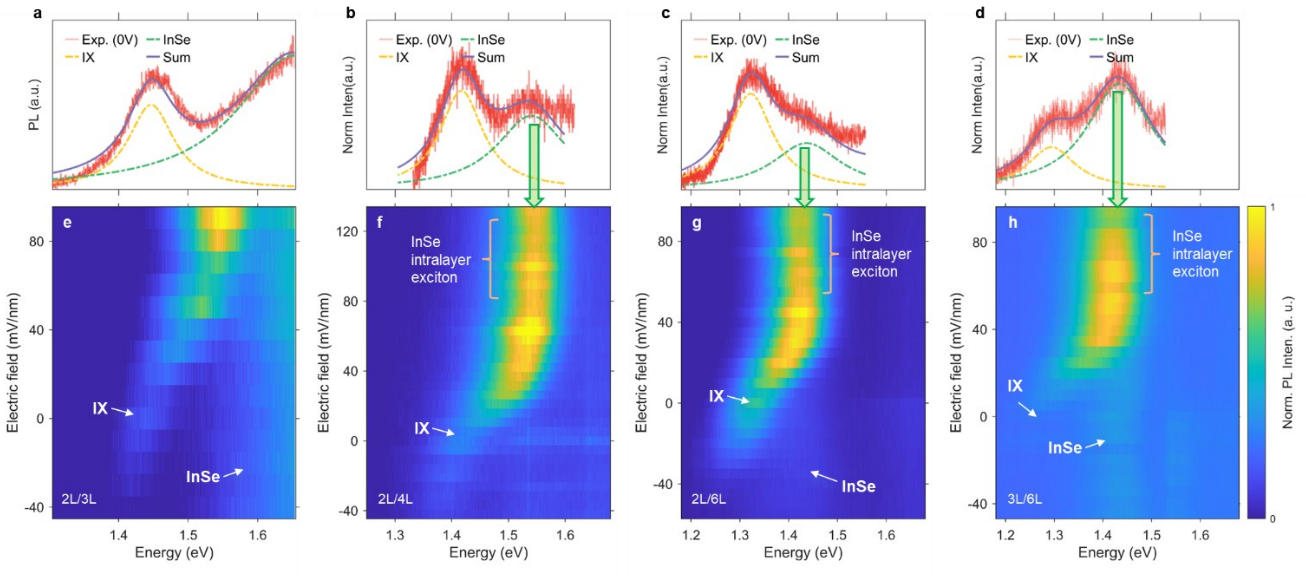

Figure S19. Evolution of IX emission with electric field for different combinations of heterostructures: 2L/3L (e), 2L/4L (f), 2L/6L (g), and 3L/6L (h). (a-d) Corresponding PL spectra at zero electric field.

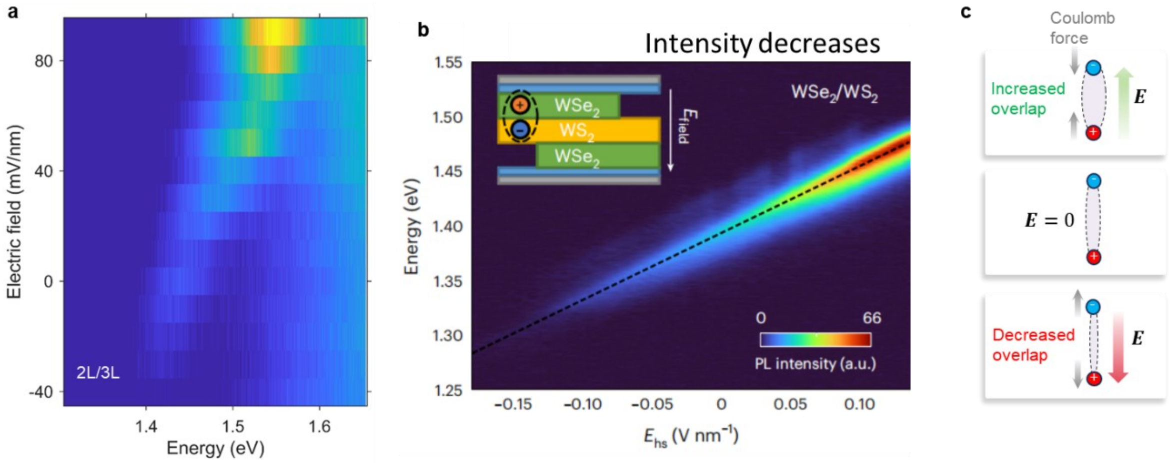

Figure S20. (a) Same image as Figure S19e. (b) Cited image from the Figure 2a in the main text of Ref. [*Nat. Mater.* **22**, 1485–1491 (2023)]. (c) Schematic illustration of PL variation with the field.

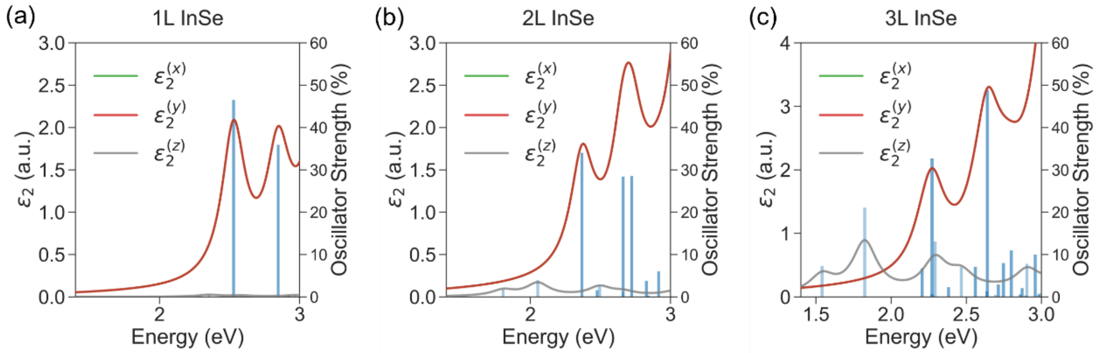

Figure S21. Optical absorption spectra and Oscillator Strength of (a-c) 1L, 2L and 3L InSe, calculated via DFT-GW-BSE.

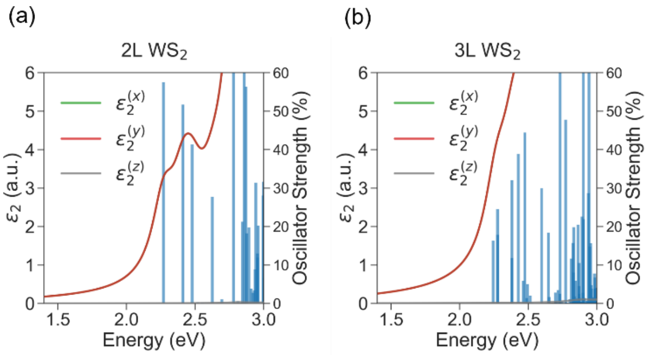

Figure S22. Optical absorption spectra and Oscillator Strength of (a-b) 2L and 3L WS_2, calculated via DFT-GW-BSE.

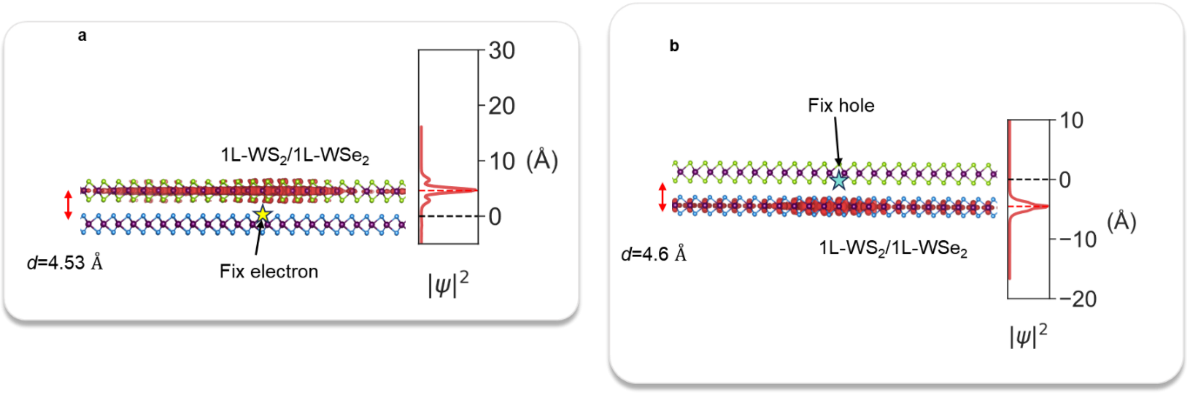

Figure S23. (**a**) The wavefunction distribution of hole in 1L-WSe_2 layer when it's formed heterostructure with 1L-WS_2 (an electron is fixed at the edge of WS_2). (**b**) The wavefunction distribution of electron in 1L-WS_2 layer when it's formed heterostructure with 1L-WSe_2 (a hole is fixed at the edge of WSe_2).

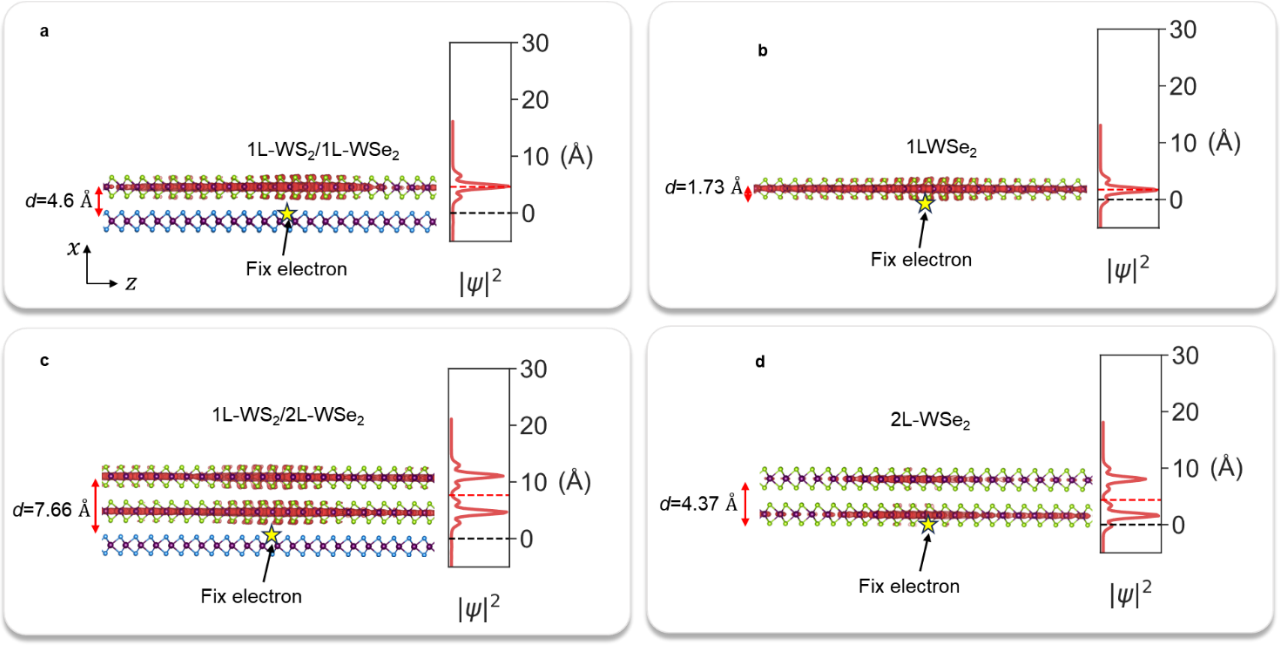

Figure S24. (**a**) The wavefunction distribution of hole in 1L-WSe_2 layer when it's formed heterostructure with 1L-WS_2 (an electron is fixed at the edge of WS_2). (**b**) The same as (**a**) but there is no 1L-WS_2 and the fixed electron is at the edge Se atom of WSe_2. (**c-d**) Same as (**a-b**) but for the 2L-WSe_2.

Supplementary Note S3. Estimation of upper limits of dipole moment

As **Figure R10** indicates, the increase of dipole moment with the increasing InSe or WS_2 layer number are not saturated. There are majorly two factors that will set the limits of the largest dipole moment we can approach:

1. The disruption of type-II alignment as the number of InSe and WS2 layers increases:

As **Figure R11a** indicates, with the layer number of InSe increased from the 2L to 5L layer, the energy difference between InSe VBM and WS_2 VBM is narrowed from 0.39 eV to 0.18 eV (according to Ref. [1]). As the experimental data shows (**Figure R11b**, directly cited from Figure 3a in [*npj 2D Mater Appl* **8**, 12 (2024).]), the difference will approach 0 at the layer number of around 18, leading to the disappearance of type-II alignment. As a result, we can roughly estimated the large dipole moment at the limits of 18-L InSe as $p_{largest} = (2.68\ enm) + (0.3\ enm) * 12 = 6.28\ enm$, where 2.68 enm is the dipole moment for 2L/6L heterostructure and for each increased layer the total dipole moment is estimated to be increased by 0.3 $e$nm (according to the increased diple moment from 2L/5L to 2L/6L).

As for WS_2, similar analyses can be applied. As **Figuer R11c** indicates, the CBM of InSe and WS_2 would approach each other with the increasing layer number of WS_2. However, the variation of CBM of WS_2 with layer number is smaller (Peak I in **Figure R11d**, which is directly cited from Figuer 3c in [*Sci Rep* **3**, 1608 (2013).] than the trend observed in InSe. Increasing WS_2 layer to the bulk would result in a very close CBM of

WS_2 to that of InSe (instead of surpass that), which would compete with the interlayer exciton transition. However, due to the lack of the further band structure data for various layer of WS2, the estimation of upper limits of dipole moment with the increasing WS_2 layer is not valid.

2. Binding energy:

The interaction between the electron and hole can be described by the Coulomb potential which is proportional to $\frac{1}{\varepsilon_r d}$ where ε_r is the relative permittivity of heterostructure (which is roughly treated as constant for the different layer combination) and d is the dipole size. Consequently, the larger dipole size would result in the weak Coulomb attraction, and thus reduce the binding enery of the exciton. In previous report (in Ref. [1]), the binding energy for 2L/3L heterostructure is estimated less than 80 meV. Since the binding enery $E_b \propto \frac{1}{d}$ where the d=1.35 nm for 2L/3L combination, and considering the binding energy should be larger than thermal energy of the environment (i.e., $k_B T \approx 7$ meV for T=77 K), the largest dipole size is thus estimated as around 15 $e$nm. Compared with band structure analyses, the binding energy analyses give a larger upper limits for dipole moment. Moreover, lowering the measurement temperature would increase the upper limits set by the binding energy.

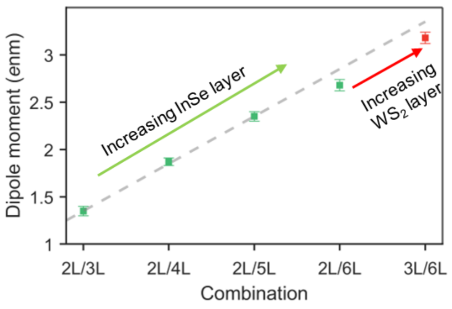

Figure S25. The evolution of dipole moment with different layer combinations. The error bars arise from the linear fitting to the Stark shifts.

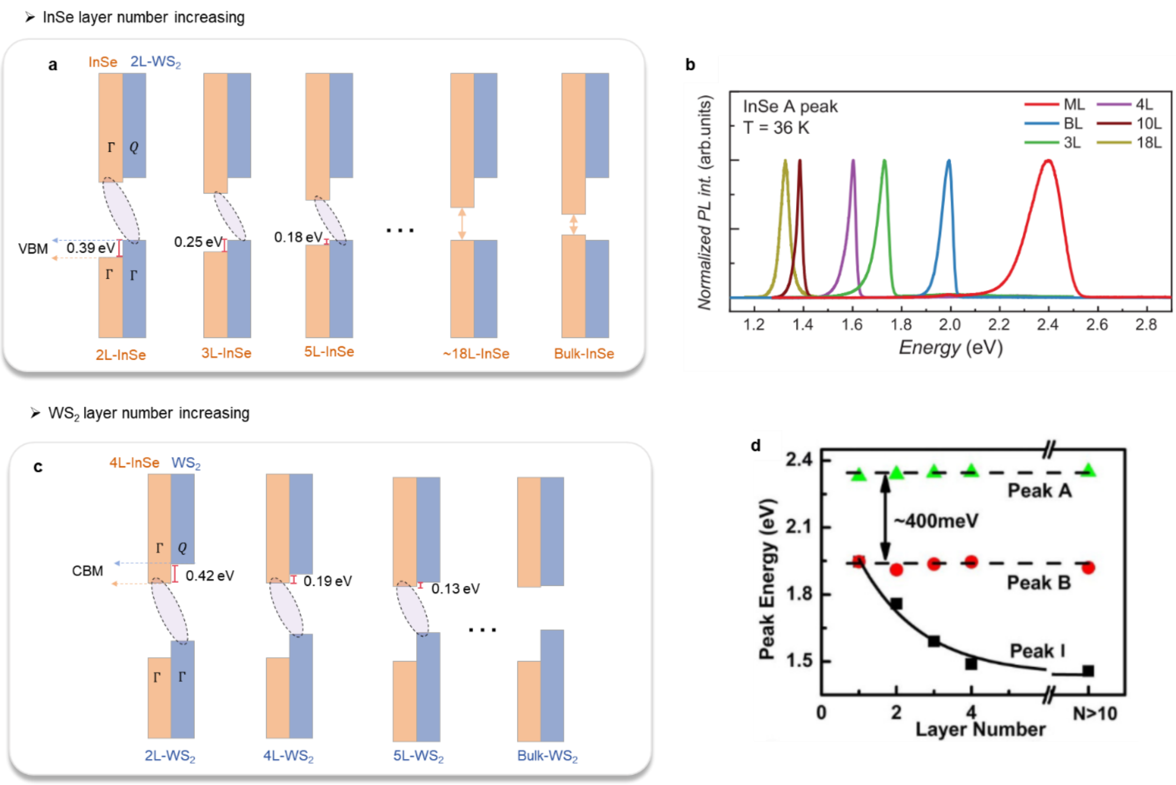

Figure S26. (**a**) Evolution of band structures of 2L-WS_2/NL-InSe heterostructure with the increasing layer number of InSe. (**b**) Measured PL spectra for InSe of various layer number (directly cited from Figure 3a in Ref. [*npj 2D Mater Appl* **8**, 12 (2024).]). (**c**) Evolution of band structures of NL-WS_2/2L-InSe heterostructure with the increasing layer number of WS_2. (**d**) The PL peak positions for WS_2 of various layer number (directly cited from Figuer 3c in Ref. [*Sci Rep* **3**, 1608 (2013).]).

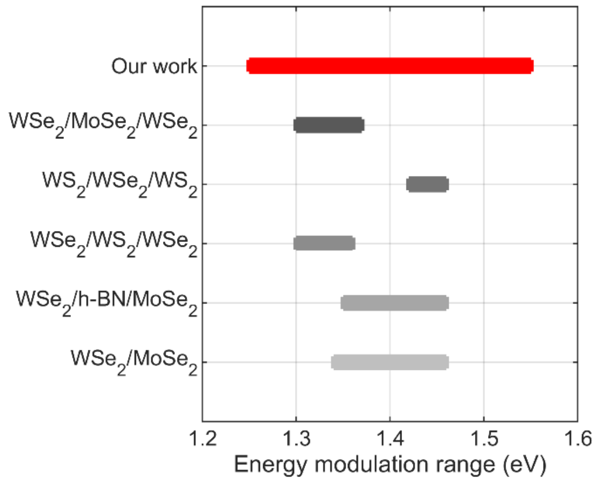

Figure S27. Comparison of tunable range of IX with both the electric- and layer-engineering. The data from bottom to top are extracted from the Refs. [11, 1, 12-14] respectively.

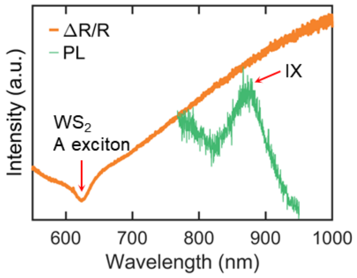

Figure S28. Measured reflection (orange curve) and PL (green curve) spectra of a representative 2L/5L heterostructu**re.**

References

1. F Mahdikhanysarvejahany, *et al.* Localized interlayer excitons in $MoSe_2$-WSe_2 heterostructures without a moire potential. *Nat. Commun.* **13**, 5354 (2022).

2. Jauregui. Electrical control of interlayer exciton dynamics in atomically thin heterostructures. *Science* **366**, 870–875 (2019).

3. PE Blöchl. Projector augmented-wave method. *Phys. Rev. B* **50**, 17953-17979 (1994).

4. G Kresse & Furthmüller J. Efficiency of ab-initio total energy calculations for metals and semiconductors using a plane-wave basis set. *Comput. Mater. Sci.* **6**, 15-50 (1996).

5. JP Perdew, Burke K & Ernzerhof M. Generalized Gradient Approximation Made Simple. *Phys. Rev. Lett.* **77**, 3865-3868 (1996).

6. S Grimme, Antony J, Ehrlich S & Krieg H. A consistent and accurate ab initio parametrization of density functional dispersion correction (DFT-D) for the 94 elements H-Pu. *J Chem Phys* **132**, 154104 (2010).

7. M Shishkin & Kresse G. Implementation and performance of the frequency-dependent *GW* method within the PAW framework. *Phys. Rev. B.* **74**, 035101 (2006).

8. AA Mostofi, Yates JR, Lee Y-S, Souza I, Vanderbilt D & Marzari N. wannier90: A tool for obtaining maximally-localised Wannier functions. *Comput. Phys. Commun.* **178**, 685-699 (2008).

9. W Hanke & Sham LJ. Many-Particle Effects in the Optical Excitations of a Semiconductor. *Phys. Rev. Lett.* **43**, 387-390 (1979).

10. K Momma & Izumi F. VESTA 3 for Three-Dimensional Visualization of Crystal, Volumetric and Morphology Data. *J. Appl. Crystallogr.* **44**, (2011).

11. Ciarrocchi, A., Unuchek, D., Avsar, A. *et al.* Polarization switching and electrical control of interlayer excitons in two-dimensional van der Waals heterostructures. *Nat. Photon.* **13**, 131–136 (2019).

12. Yu, L., Pistunova, K., Hu, J. *et al.* Observation of quadrupolar and dipolar excitons in a semiconductor heterotrilayer. *Nat. Mater.* **22**, 1485–1491 (2023).

13. Li, W., Hadjri, Z., Devenica, L.M. *et al.* Quadrupolar–dipolar excitonic transition in a tunnel-coupled van der Waals heterotrilayer. *Nat. Mater.* **22**, 1478–1484 (2023).

14. Xie, Y., Gao, Y., Chen, F., Wang, *et al.* Bright and dark quadrupolar excitons in the WSe_2/$Mose_2$/WSe_2 heterotrilayer. *Phys. Rev. Lett.*, ***131***, 186901 (2023).
